# Preferential Reactivity of Dipeptidyl Peptidase-IV Inhibitor-Associated Bullous Pemphigoid Autoantibodies to the Processed Extracellular Domains of BP180

**DOI:** 10.3389/fimmu.2019.01224

**Published:** 2019-05-29

**Authors:** Yosuke Mai, Wataru Nishie, Kentaro Izumi, Hiroshi Shimizu

**Affiliations:** Department of Dermatology, Hokkaido University Graduate School of Medicine, Sapporo, Japan

**Keywords:** bullous pemphigoid, dipeptidyl peptidase-IV inhibitor, dipeptidyl peptidase-IV inhibitor-associated bullous pemphigoid, autoantibodies, IgG subclass, BP180, BP230, autoimmune disease

## Abstract

Bullous pemphigoid (BP) is a common autoimmune blistering disease in which autoantibodies target the hemidesmosomal components BP180 and/or BP230 in basal keratinocytes. In BP, 80 to 90% of autoantibodies target the juxtamembranous extracellular non-collagenous 16th A (NC16A) domain of BP180. Recently, the administration of dipeptidyl peptidase-IV inhibitors (DPP4i), which are widely used as antihyperglycemic drugs, has been recognized to be a causative factor for BP. DPP4i-associated BP (DPP4i-BP) autoantibodies tend to target epitopes on non-NC16A regions of BP180, and the pathomechanism for the development of the unique autoantibodies remains unknown. To address the characteristics of DPP4i-BP autoantibodies in detail, we performed epitope analysis of 18 DPP4i-BP autoantibodies targeting the non-NC16A domains of BP180 using various domain-specific as well as plasmin-digested polypeptides derived from recombinant BP180. Firstly, Western blotting showed that only one DPP4i-BP serum reacted with the epitopes on the intracellular domain of BP180, and no sera reacted with the C-terminal domain of the molecule. In addition, only 2 DPP4i-BP sera reacted with BP230 as determined by enzyme-linked immunosorbent assay. Thus, DPP4i-BP autoantibodies were found to mainly target the non-NC16A mid-portion of the extracellular domain of BP. Interestingly, Western blotting using plasmin-digested BP180 as a substrate revealed that all of the DPP4i-BP sera reacted more intensively with the 97-kDa processed extracellular domain of BP180, which is known as the LABD97 autoantigen, than full-length BP180 did. All of the DPP4i-BP autoantibodies targeting the LABD97 autoantigen were IgG1, and IgG4 was observed to react with the molecule in only 7 cases (38.9%). In summary, the present study suggests that IgG1-class autoantibodies targeting epitopes on the processed extracellular domain of BP180, i.e., LABD97, are the major autoantibodies in DPP4i-BP.

## Introduction

Bullous pemphigoid (BP) is a common autoimmune blistering disease in which autoantibodies target the hemidesmosomal components BP180 and/or BP230 in basal keratinocytes (1). In BP, almost 90% of autoantibodies target the juxtamembranous extracellular non-collagenous 16th A (NC16A) domain of BP180 (2). Recently, the administration of dipeptidyl peptidase-IV inhibitors (DPP4i), which are widely used as antihyperglycemic drugs, is recognized to be a causative factor for BP (3). In 2016, a strong relationship between the onset of BP under DPP4i exposure was reported from the France Pharmacovigilance Database (4). Thereafter, retrospective case-control studies from Finland, (5) Switzerland, (6) France, (6) Israel (7), and Japan (the Japanese Adverse Drug Event Report Database) (8) also confirmed that the administration of DPP4i, especially vildagliptin, was associated with increased risk of BP development. In addition, based on the National Healthcare Insurance Agency of France, the observed frequencies of DPP4i intake in the BP population are higher than those in the general population (9). Although the increased risk of BP development after the use of DPP4i has been intensively studied, the mechanisms whereby DPP4i administration causes BP development remains to be elucidated.

We and other groups have recently reported that Japanese patients with DPP4i-associated BP (DPP4i-BP) may show unique clinical and immunological characteristics (10–14). Clinically, DPP4i-BP patients tend to show a less severe erythematous phenotype than typical non-DPP4i BP patients show. In terms of the Bullous Pemphigoid Disease Area Index (BPDAI) (15) a commonly used disease severity score for BP, patients with typical BP do not differ significantly from patients with DPP4i-BP in terms of erosion/blister scores, whereas patients with DPP4i-BP have significantly less severe erythema/urticaria than patients with typical BP have. Immunologically, DPP4i-BP autoantibodies preferentially target the mid-portion of the extracellular region of BP180 without reactivity toward the NC16A domain (10). Interestingly anti-BP180 NC16A autoantibodies may be produced during the clinical course of DPP4i-BP as a result of epitope spreading (13, 14). After the production of anti-BP180 NC16A autoantibodies, clinical manifestations of DPP4i-BP may resemble those of typical non-DPP4i-BP (13, 14). Thus, anti-BP180 NC16A autoantibodies may be observed also in DPP4i-BP.

In this study, we collected 18 cases of “pure” DPP4i-BP in which autoantibodies did not target the NC16A domain of BP180. As a result, it has been revealed that DPP4i-BP autoantibodies are IgG1-class autoantibodies which target epitopes on the non-NC16A processed extracellular domains of BP180.

## Materials and Methods

### Patient Characteristics

BP was diagnosed based on clinical, histopathological, and immunological findings (1). The clinical and immunological characteristics of the DPP4i-BP cases in this study are listed in Table 1 and summarized in Table 2. Direct immunofluorescence study showed positive results for IgG and/or C3 deposition at the basement membrane zone in all cases. The mean age was 78.8 and ranged from 57 to 93. The female/male ratio was 6:12. The mean index value of full-length BP180 ELISA was 108.2 and ranged from 60.9 to 171.7 (cutoff < 4.64). None of our cases was positive for BP180 NC16A by chemiluminescent enzyme immunoassay. BPDAI scores for blister/erosion, urticaria/erythema, and total mucosa are summarized in Table 1. Vildagliptin was the most common DPP4i (38.9%), followed by teneligliptin (33.3%), and Sitagliptin (27.8%) (Table 3).

**Table 1:** List of DPP4i-BP cases.

**No**	**Age**	**Sex**	**Full-length BP180**	**BP180 NC16A**	**BP230**	**DPP4i**	**DIF**	**ssIIF**	**Delay after BP onset**	**BPDAI**
1	55–60	Male	94.4	(–)	2.3	Teneligliptin	IgG, C3	roof side	–	–
2	75–80	Male	86.6	(–)	1.5	Vildagliptin, Teneligliptin, Sitagliptin	IgG, C3	roof side	7 months	–
3	90–95	Male	150.6	(–)	2.3	Anagliptin	IgG, IgA, IgM, C3	roof side	7 months	52–7–0
4	90–95	Female	130.6	(–)	3.4	Vildagliptin	IgG, C3	roof side	2 months	36–NA–5
5	70–75	Male	103.8	(–)	1.7	Teneligliptin, Vildagliptin	IgG, C3	roof side	6 months	–
6	80–85	Male	102.3	(–)	4.7	Vildagliptin	IgG, C3	roof side	2 months	–
7	65–70	Male	60.93	(–)	0.4	Sitagliptin	IgG, C3	roof side	3 months	–
8	85–90	Female	109.9	(–)	1.7	Teneligliptin	IgG, IgM, C3	roof side	2 months	total 30
9	80–85	Female	171.7	(–)	1.1	Omarigliptin	C3	roof side	2 months	–
10	90–95	Female	98.9	(–)	20.3	Alogliptin, Sitagliptin	IgG, C3	roof side	2 months	–
11	85–90	Male	113.2	(–)	1.9	Teneligliptin	IgG, C3	roof side	1 month	7–6–0
12	55–60	Female	139	(–)	2.6	Vildagliptin	IgG, C3	roof side	2 months	9–7–15
13	70–75	Male	81.7	(–)	4.1	Vildagliptin, Sitagliptin	IgG, IgA, C3	roof side	–	13–0–0
14	85–90	Male	111.2	(–)	3.3	Alogliptin, Linagliptin	IgG, IgA, IgM, C3	roof side	2 months	38–0–0
15	70–74	Male	103.7	(–)	14.3	Vildagliptin	IgG, C3	roof side	3 months	46–3–0
16	75–80	Male	84.5	(–)	2.5	Teneligliptin	IgG C3	roof side	2 weeks	–
17	50–60	Female	79.5	(–)	2.3	Anagliptin, Linagliptin	IgG, C3	roof side	8 months	–
18	90–95	Male	125.4	(–)	3.7	Sitagliptin, Linagliptin	IgG, C3	roof side	2 weeks	–

*Full-length BP180 ELISA: Index values, cutoff <4.64. BP230ELISA: Index values, cutoff < 9.0. NA, means not available*.

**Table 2:** Summary of patient attributes.

Age, mean (range), years	78.8 (57–93)
Female/male, *n*; sex ratio	6/12
Full-length BP180 ELISA, mean (range), index values	108.2 (60.9–171)
BP180 NC16a, positive rate, *n* (%)	0 (0%)
BP230 ELISA, mean (range), index values	4.2 (1.1–20.3)
BP230 ELISA, positive rate, *n* (%)	2 (11.1%)

**Table 3:** DPP4i use.

Vildagliptin	7 (38.9%)
Teneligliptin	6 (33.3%)
Sitagliptin	5 (27.8%)
Linagliptin	3 (16.7%)
Alogliptin	2 (11.1%)
Anagliptin	2 (111%)
Omarigliptin	1 (5.6%)

### Preparation of Recombinant Proteins

**Figure 1:**
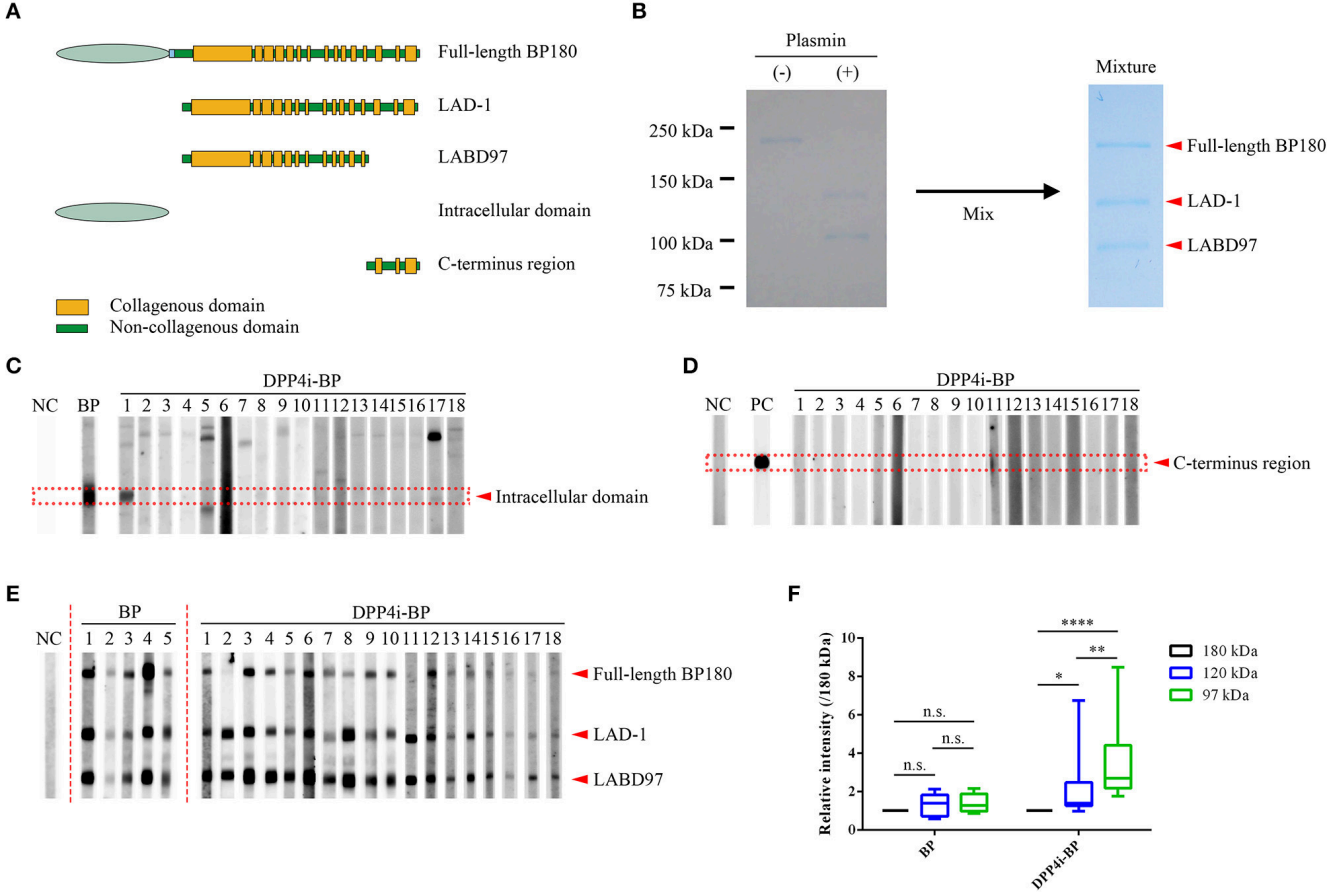
Epitope mapping of DPP4i-BP autoantibodies. **(A)** Schematic of recombinant proteins. **(B)** Coomassie Blue staining using a mixture of substrates including the full-length recombinant BP180 and plasmin-digested BP180 proteins. **(C–E)** Western blots using the intracellular domain of BP180 **(C)**, C-terminal regions of BP180 **(D)**, and a mixture of full-length recombinant BP180 and plasmin-digested BP180 proteins **(E)**. Relative intensities of 180-kDa, 120-kDa, and 97-kDa bands in BP and DPP4i-BP **(F)**. Data were analyzed using two-way analysis of variance, followed by Tukey's multiple comparison test. *p*-values are indicated as *0.01 < *p* < 0.05, **0.001 < *p* < 0.01, and ****p < 0.0001. n.s., Not significant; NC, Negative control using healthy individual sera; PC, Positive control using anti-FLAG antibody.

Full-length human BP180 recombinant protein (Met^1^ – Pro^1497^) and polypeptides corresponding to the intracellular domain (Met^1^ to Trp^467^) and the C-terminus region (Leu^1281^ – Pro^1497^) of BP180 were produced using the Flp-In 293 system (Invitrogen, CA) as previously reported (10). Processed BP180 extracellular fragments of 120-kDa and 97-kDa forms, which are known as LAD-1 and LABD97, respectively, were generated by limited plasmin digestion of the full-length recombinant BP180 protein (10). Schematics of the recombinant proteins and the plasmin-digested proteins are given in Figure 1A. Mixture substrate samples of full-length BP180, LAD-1, and LABD97 were used for Western blotting, of which even doses were confirmed by Coomassie Blue staining (Figure 1B).

### Immunofluorescence Study

For indirect immunofluorescence study using 1 M NaCl-split skin, normal human skin was incubated in 1 M NaCl solution for 48 h at 4°C. Thereafter, the skin was mounted and snap-frozen in OCT compound (Thermo Fisher Scientific, MA), and 5-μm cryosections were prepared. The sections were then incubated with patients' sera (dilution 1:10–20) for 40 min at 37°C. After washing with PBS 3 times, the sections were incubated with FITC-conjugated antibodies to human IgG (dilution 1:100) (Dako Cytomation, Denmark) for 30 min at 37°C.

### Western Blotting

Protein samples were separated by SDS-PAGE electrophoresis using 7 or 10% SDS-polyacrylamide gel. The gels were transferred to nitrocellulose membranes (Bio Rad, CA). The membranes were blocked for 30 min at room temperature with 2% skimmed milk in TBS. After incubation with 1:200 diluted patient serum with 2% skimmed milk in TBS for 1 h at room temperature, horseradish peroxidase-conjugated secondary anti-human IgG (1:1,000) (Dako Cytomation, Denmark), IgG1 (1:500) (Thermo Fisher Scientific, MA), or IgG4 (1:500) (Thermo Fisher Scientific, MA) antibodies in the same buffer were reacted at room temperature for 1 h. Signals were visualized with Clarity Western ECL Substrate (Bio Rad, CA). Each protein band was quantified using Fiji (16). Relative intensities were calculated for each band based on the intensity of the 180-kDa bands. Case No.11 was excluded since the intensity of the 180-kDa band was faint.

### Enzyme-Linked Immunosorbent Assay (ELISA)

ELISA using full-length BP180 recombinant proteins was performed as previously described, with a minor modification (10). Briefly, 96-well plates (Thermo Fisher Scientific, MA) were coated with 1 μg/well of the recombinant proteins in 50 mM carbonate buffer pH 9.5 and then blocked with Blocking Reagent for ELISA (Roche, Swiss) for 2 h at room temperature. Patient sera were diluted to 1:100 and incubated for 1 h at room temperature. After washing, the plates were incubated with 1:500 diluted horseradish peroxidase-conjugated anti-human IgG antibody for 1 h at room temperature. Subsequently, the plates were reacted with a substrate solution, 3, 3′, 5, 5′tetramthylbenzidine dihydrochloride single solution (Thermo Fisher Scientific, MA). After color development, the reaction was stopped with the addition of 0.12 N hydrochloric acid, and absorbance was measured at 450 nm by a microplate reader with the correlation wavelength set at 620 nm (Tecan Austria GmbH, Austria). To detect anti-BP230 autoantibodies, BP230 ELISA (MESACUP BP230 ELISA kit, MBL, Japan) was performed according to the manufacturer's instructions.

### Statistical Analysis

Statistical analysis was performed using GraphPad Prism 6 (GraphPad Software, USA). Data were analyzed using two-way analysis of variance, followed by Tukey's multiple comparison test. *p*-values are indicated as ^*^0.01 < *p* < 0.05, ^**^0.001 < *p* < 0.01, and ^****^p < 0.0001.

## Results

### Preferential Reactivity of DPP4i-BP Autoantibodies to the Processed Extracellular Domain of BP180

Firstly, we performed Western blotting using the recombinant intracellular and C-terminal proteins of BP180 (Figures 1C,D). Only one case had autoantibodies targeting the intracellular region of BP180, and no cases had autoantibodies targeting both fragments (Figures 1C,D). In addition, autoantibodies targeting BP230 were only detected in two cases (11.1%) by BP230 ELISA (cutoff < 9.0) (Tables 1, 2). We also confirmed that there were no IgG autoantibodies targeting the dermal side of the artificial blisters in 1 M NaCl-split skin indirect immunofluorescence (Supplemental Figure 1).

To identify the epitopes of IgG autoantibodies in DPP4i-BP, we performed Western blotting using mixture samples of full-length BP180 and plasmin-digested 120-kDa (LAD-1) and 97-kDa (LABD97) fragments (Figure 1E). IgG autoantibodies of all cases except for case No.11 reacted to the full-length BP180 (Figure 1E), which is mostly consistent with the result of full-length BP180 ELISA (Table 1). Interestingly, IgG autoantibodies showed more intense reactivity to LABD97 than to LAD-1 or full-length BP180 (Figures 1E,F). In contrast, the autoantibodies in typical non-DPP4i BP reacted almost equally to each band (Figures 1E,F). Since none of the cases reacted with the NC16A domain of BP180, all of the DPP4i-BP autoantibodies tested in this study showed preferential reactivities toward the processed extracellular domain of BP180.

### IgG1 Autoantibodies to LABD97 Are Major Autoantibodies in DPP4I-BP

**Figure 2:**
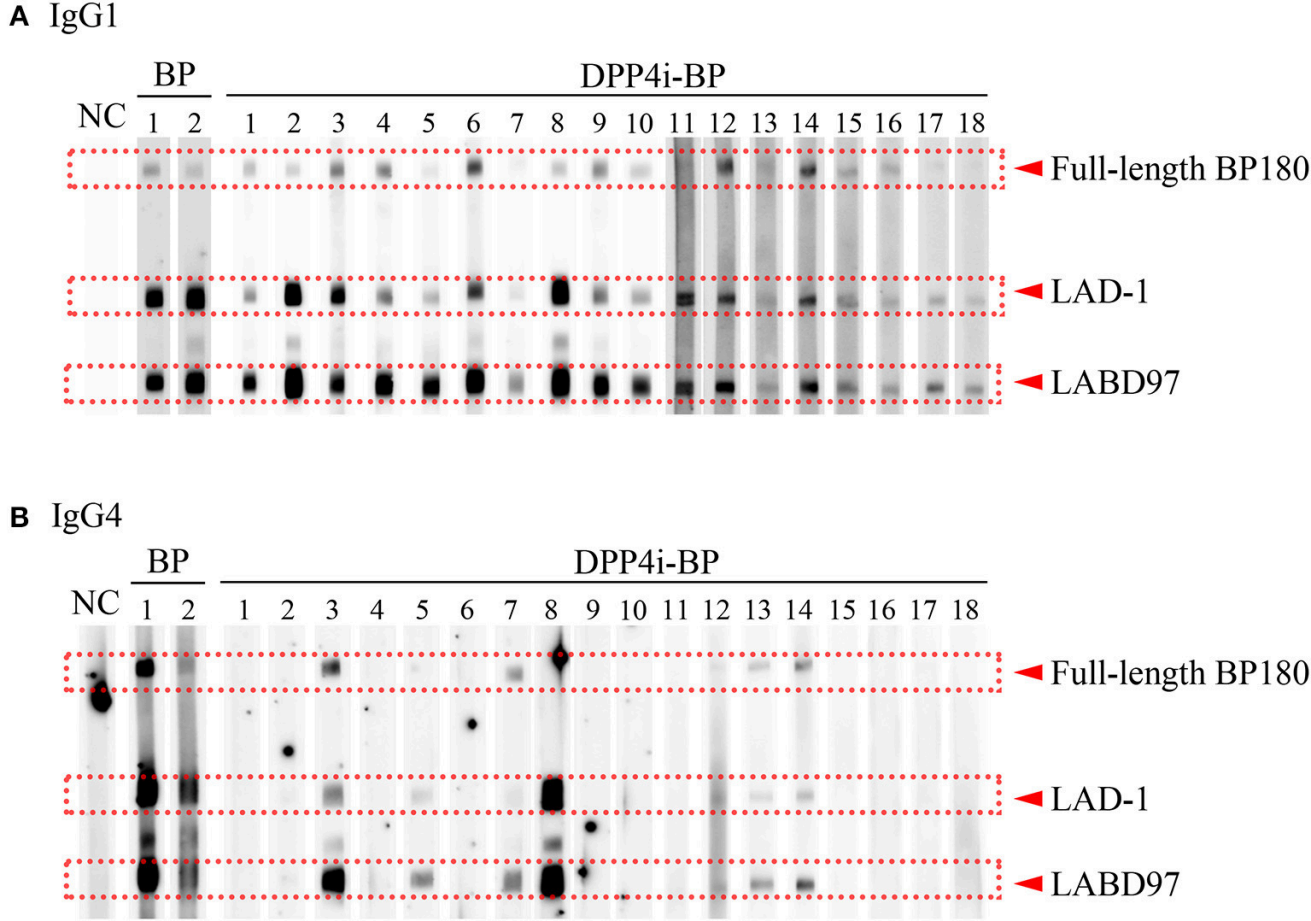
IgG subclass analysis of DPP4i-BP. Detection of IgG1 **(A)** and IgG4 **(B)** autoantibodies by Western blot using a mixture of full-length recombinant BP180 and plasmin-digested BP180 proteins.

It is known that both IgG1 and IgG4 are the predominant autoantibodies in BP (17). Therefore, we evaluated IgG1 and IgG4 autoantibody reactivities to the full-length BP180 and the plasmin-digested LAD-1 and LABD97 fragments of BP180. Although all cases had IgG1 autoantibodies targeting each form of BP180, it should be noted that significant reactivities to LABD97 were observed (Figure 2). In contrast, 7 of 18 cases (38.9%) had IgG4 autoantibodies targeting various forms of BP180 (Figure 2). These findings suggest that IgG1 autoantibodies to LABD97 are major autoantibodies in DPP4i-BP.

## Discussion

In this study, we analyzed autoantibodies of 18 DPP4i-BP cases without reactivity to the NC16A domain of BP180. Consistent with our previous study using small numbers of DPP4i-BP cases, (10) all of the autoantibodies showed positive reactivity to the processed extracellular domain of BP180. Interestingly, all of the DPP4i-BP autoantibodies tested in this study showed preferential reactivity to LABD97. A similar characteristic of autoantibodies can be found in another autoimmune blistering skin disease, linear IgA bullous dermatosis. In linear IgA bullous dermatosis, autoantibodies preferentially react with neoepitopes that selectively develop on LAD-1 and LABD97, and they do not react with full-length BP180 (18, 19). BP180 can be processed by several proteases under physiological or pathological conditions, Franzke et al. (20), Hofmann et al. (21) and the N-terminal deletion resulting from cleavage within the NC16A domain of BP180 results in the production of LAD-1 (20). In addition, further C-terminal deletion produces LABD97 (21). This processing of BP180 may induce conformational changes on the molecule, which also may induce new epitopes distinct from those on the native full-length form of BP180 (22–25). Several studies have shown that antibodies targeting the 15th collagenous domain or its neighboring regions in BP180 preferentially react with LAD-1 and LABD97 (22, 23). In addition, our previous study revealed that the C-terminus processing of BP180 induces neoepitopes on the 15th collagenous domain of BP180 (24). Thus, not only does cleavage within the NC16A domain cause neoepitopes to develop on the 15th collagenous domain of BP180, but so does C-terminal processing (25). Although fine epitope mapping of DPP4i-BP autoantibodies will be necessary in future studies, the preferential reactivity of DPP4i-BP autoantibodies to LABD97 may suggest the possibility that putative epitopes may exit on the 15th collagenous domain or in its neighboring regions.

The present study also revealed that all of the DPP4i-BP autoantibodies which preferentially targeted the LABD97 fragment of BP180 were IgG1. In contrast, IgG4-subclass autoantibodies were only observed in 38.9% of our cases. These results indicate that IgG1 autoantibodies targeting epitopes on LABD97 are the main autoantibodies in DPP4i-BP. This finding was unexpected, because it is well-known that IgG1 BP autoantibodies are able to activate the complement pathway associated with the development of inflammation, (26) whereas DPP4i-BP tends to show a non-inflammatory phenotype associated with scant urticarial erythema and fewer infiltrating eosinophils than those of non-DPP4i BP, despite activating the complement at dermal-epidermal junctions (10–14). Thus, the predominance of IgG1 autoantibodies in DPP4i-BP stands in contrast to the previous concept of BP pathogenesis (26). Although further studies are necessary to resolve this issue, the present study indicates that complement activation is insufficient to induce inflammation in DPP4i-BP.

The pathogenicity of IgG4 is controversial. An experimental mouse model showed that IgG4 plays a protective role against the development of BP (27). In contrast, it is experimentally shown that IgG4 BP autoantibodies have the capacity to induce leucocyte-dependent dermal-epidermal separation (28). In addition, BP cases with only IgG4 autoantibodies have been reported (29). As this IgG4-type BP shows a non-inflammatory phenotype, IgG4 autoantibodies are assumed to play complement-independent roles in BP development, such as a role in BP180 depletion in keratinocytes, the Fc-dependent activation of neutrophils, or leucocyte-dependent dermal-epidermal separation (26, 28, 30). In our subclass analysis of DPP4i-BP, IgG4 autoantibodies preferentially reacted to LABD97, similar to IgG1 autoantibodies. However, the positive rates for IgG4 autoantibodies to LABD97 and full-length BP180 were lower than those of IgG1 autoantibodies, especially to full-length BP180. This discrepancy may suggest that the development of IgG1 autoantibodies might precede IgG4 autoantibody development in DPP4i-BP, although other possibilities cannot be ruled out.

## Concluding Remarks

In conclusion, major epitopes of DPP4i-BP were located in the mid-portion of the extracellular domain, and these epitopes can be presented on LABD97. All such autoantibodies were in the IgG1 class. The unique immunological characteristics of DPP4i-BP autoantibodies may help us understand the pathological mechanism behind the development of the disease.

## Ethics Statement

This report on a single patient complies with the Declaration of Helsinki. The patient gave written informed consent for publication. This study was approved by the institutional review board of Hokkaido University (institutional review board number: 016-0061).

## Author Contributions

YM, WN, and KI drafted the paper and collected the clinical data. HS supervised the writing of the manuscript.

### Conflict of Interest Statement

The authors declare that the research was conducted in the absence of any commercial or financial relationships that could be construed as a potential conflict of interest.

## Supplementary Material

Supplemental Figure 1 Indirect Immunofluorescence Study Using 1 M NaCl-split Skin. NC, Negative control using healthy individual sera.

## Abbreviations

BP: bullous pemphigoid
NC16A: the 16th non-collagenous domain
DPP4i: dipeptidyl peptidase-IV inhibitor(s)
DPP4i-BP: dipeptidyl peptidase-IV inhibitor-associated bullous pemphigoid
ELISA: enzyme-linked immunosorbent assay
BPDAI: bullous pemphigoid disease area index.
