# Supplementary figures and images for: Preferential Reactivity of Dipeptidyl Peptidase-IV Inhibitor-Associated Bullous Pemphigoid Autoantibodies to the Processed Extracellular Domains of BP180
Source: Front Immunol. 2019 May 29;10:1224. doi: 10.3389/fimmu.2019.01224 (PMC6549357; doi:10.3389/fimmu.2019.01224)

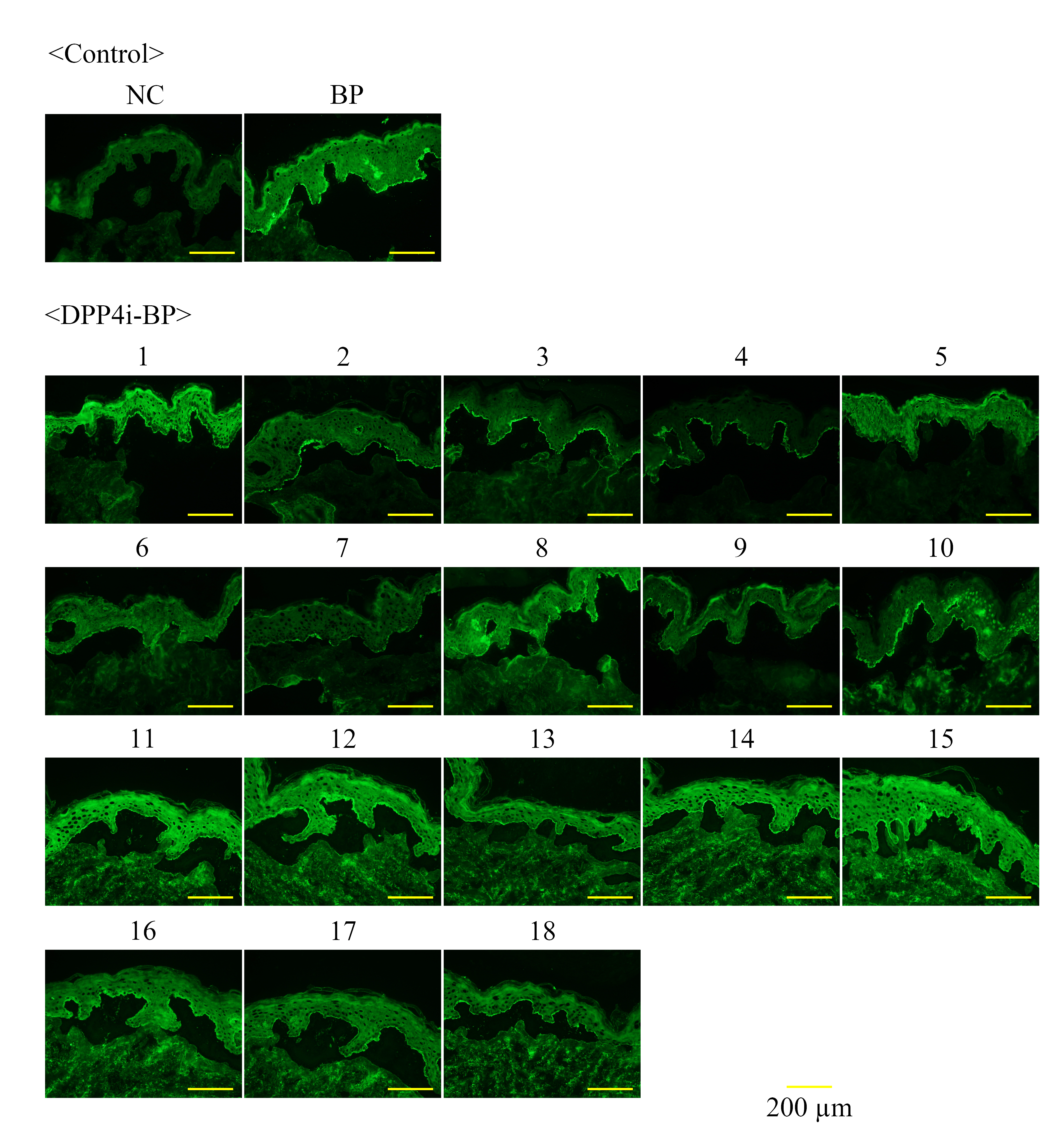

Supplement: Supplemental Figure 1 — Indirect Immunofluorescence Study Using 1 M NaCl-split Skin. NC, Negative control using healthy individual sera. [file Image_1.TIF]
